# Effect of Coated Inorganic Micro-Minerals on Growth, Mineral Retention, and Intestinal Health in Juvenile American Eels Under a Commercial RAS

**DOI:** 10.3390/ani16020324

**Published:** 2026-01-21

**Authors:** Xiaozhao Han, Deying Ma, Yichuang Xu, Shaowei Zhai

**Affiliations:** 1 Fisheries College, Jimei University, Xiamen 361021, China; 202414908011@jmu.edu.cn (X.H.); sdmadeying@163.com (D.M.); xuyichuang@jmu.edu.cn (Y.X.); 2 Engineering Research Center of the Modern Technology for Eel Industry, Ministry of Education, Xiamen 361021, China

**Keywords:** *Anguilla rostrata*, antioxidant capacity, intestinal microbiota, metabolomics, commercial aquaculture

## Abstract

Micro-minerals are essential for fish, but traditional inorganic micro-minerals (IMM) have low bioavailability. This study evaluated coated inorganic micro-minerals (CIMM) in juvenile American eels under commercial recirculating aquaculture system (RAS) conditions. Three experimental groups (*n* = 3 tanks per group, stocking density: 138 fish/m^3^) were fed basal diets supplemented for 56 days with: 1000 mg/kg IMM (IMM group, providing Cu 7, Fe 200, Mn 30, Zn 70, I 1.6, Se 0.4, and Co 1.2 mg/kg diet), 1000 mg/kg CIMM (CIMM group I), or 500 mg/kg CIMM (CIMM group II). Compared to the IMM group, the CIMM group I demonstrated significantly enhanced growth performance, with the specific growth rate increasing by approximately 31.14%, higher whole-body content and retention of minerals (Ca, P, Cu, Fe, Mn, Zn), and superior intestinal health, as reflected by significantly increased activities of digestive enzymes (amylase and lipase), enhanced antioxidant capacity (elevated SOD and CAT, reduced MDA), and improved morphology (villi length and muscular thickness), an altered intestinal microbiota (increased relative abundance of Firmicutes and reduced relative abundance of Proteobacteria), and significant metabolomic alterations in purine metabolism and linoleic acid metabolism. The CIMM group II maintained growth performance, with no significant difference in WGR and SGR compared to the IMM group, while still showing significant improvements in feed intake and mineral retention (P, Cu, Fe, Zn), and antioxidant capacity. Collectively, this study not only confirms the efficacy of CIMM in commercial RAS but also reveals that the supplementation level previously shown to be effective in the laboratory (50% CIMM) is insufficient under commercial farming conditions, implying that the dietary micro-mineral requirements for juvenile American eels in commercial RAS may be higher than those established in laboratory settings.

## Simple Summary

Traditional inorganic micro-minerals can damage nutrients in fish feed and are poorly absorbed by fish. If these minerals are coated with a protective layer, their reaction with feed nutrients during the fish’s digestion can be reduced. This helps solve the above-mentioned problems and promotes fish growth and health. This study was conducted in a commercial recirculating aquaculture system (RAS) to test the effects of adding traditional vs. coated inorganic micro-minerals to feed on juvenile American eels. The assessment focused on their growth, body composition, mineral absorption, and intestinal health. The results showed that coated inorganic micro-minerals performed much better than traditional ones in all these aspects (i.e., growth and intestinal health), so using such coated micro-minerals in commercial culture conditions can significantly benefit juvenile American eels.

## 1. Introduction

Micro-minerals, including iron (Fe), zinc (Zn), copper (Cu), manganese (Mn), selenium (Se), iodine (I), cobalt (Co) and others, are essential nutrients that organisms cannot synthesize independently. Despite being required only in trace amounts, these minerals are crucial for the growth, development, and maintenance of normal intestinal morphology and structure in fish due to their fundamental roles as enzymatic cofactors, structural components, and hormonal constituents [1,2,3,4]. For instance, zinc supports serum alkaline phosphatase activity and vertebral mineralization; iron is fundamental for hemoglobin synthesis; copper and manganese activate different forms of the antioxidant enzyme superoxide dismutase; selenium is a key component of glutathione peroxidase; iodine is essential for thyroid hormone synthesis; and cobalt is the central atom of vitamin B12 [4]. If fish lack such micro-minerals, their growth will be significantly inhibited, the intestinal barrier function will be damaged, and their susceptibility to diseases will increase [5,6,7,8]. To meet the nutritional requirements of fish, the addition of micro-minerals to feed has become a necessary measure in aquaculture, and selecting an efficient form of micro-mineral supplementation is one of the prerequisites for ensuring the effectiveness of aquaculture.

Inorganic micro-minerals (IMM) are predominantly found in the form of inorganic salts and are extensively utilized due to their cost-effectiveness. However, the absorption efficiency of IMMs is low, primarily due to the dissociation of their ionic bonds during digestion, which enables interactions with dietary anions (such as oxalate, phosphate, phytate, and carbonate), forming insoluble complexes that cannot be absorbed [9]. Moreover, the oxidative properties of IMMs allow them to react readily with lipids, vitamins and other nutrients in the feed, consequently diminishing the nutritional value of the diet [10,11]. To address the limitations of IMM, organic micro-minerals (OMM) (e.g., amino acid-chelated or protein-complexed forms) have been developed and applied. Although OMMs can reduce antagonistic reactions with other feed components and partially improve absorption efficiency [12,13], they still have limitations such as oxidizing nutrients in feed [14], inconsistent effects on fish growth [15,16], and relatively high costs [12], making them difficult to be widely promoted in commercial culture conditions.

Coating technology, commonly applied in feed additives, creates a protective layer (digestible carbohydrates matrix) that isolates micro-minerals from dietary components during gastrointestinal transit, effectively preventing their interaction with digestive anions to reduce insoluble complex formation and thereby improving micro-minerals utilization [11,17]. In addition, this physical separation also diminishes oxidative interactions between micro-minerals and nutrients in the feed. Overall, coated inorganic micro-minerals (CIMM) have been shown to promote growth and intestinal health in broilers [12,14,18,19], weaning piglets [20], and growing sheep [21]. Research on CIMM in aquaculture remains in its infancy, with the only studies to date that have been conducted in grass carp (*Ctenopharyngodon idella*) and American eel (*Anguilla rostrata*) consistently demonstrating superior efficacy over IMM [9,22].

Eels are high-value fish species with considerable economic significance in global freshwater aquaculture and international trade [23]. An analysis of 2020–2022 data demonstrated China’s dominance in the global eel trade, accounting for 60.17% of the global domestic supply (averaging 172,000 tonnes annually) and 69.7% of global exports (approximately 97,000 tonnes annually), establishing it as the core supplier [24]. The American eel predominates Chinese eel aquaculture at 74% of production and thus was selected as the experimental subject in this study [24]. Previous research under laboratory conditions revealed that CIMM conferred superior growth performance and intestinal health upon juvenile American eels compared to IMM, despite a 50% reduction in supplementation levels [9]. The same study also indicated that juvenile American eels fed CIMM performed comparably to, or even better than, those fed OMM. However, this experiment was performed at the small, laboratory scale that does not adequately reflect actual commercial production, in aspects such as water quality conditions, operational disturbances, and stocking density. Therefore, to assess the practical efficacy of CIMM, this study was conducted within a commercial recirculating aquaculture system (RAS). Due to the practical difficulty in sourcing a sufficient quantity of juvenile eels with uniform size and health status under commercial farming conditions to meet the requirements of a high-replication experiment, the present trial was designed to compare CIMM against the traditional IMM. Consequently, a direct evaluation against OMM under these commercial conditions was not undertaken in the present study. The study aimed to evaluate the effects of CIMM on the growth performance, mineral retention, and intestinal health of juvenile American eels in a commercial RAS, and to verify whether the 50% reduction in CIMM supplementation, previously effective under controlled laboratory conditions, would maintain its efficacy in a commercial farming environment.

## 2. Materials and Methods

### 2.1. Experimental Design and Diets

The CIMM used in this study was the IMM encapsulated with a digestible carbohydrate matrix. Three experimental diets were formulated by supplementing the basal diet with micro-minerals premixes. The formulation and composition of experimental diets are shown in Table 1. The IMM group was supplemented with an IMM premix at a concentration of 1000 mg/kg, while the CIMM group I and CIMM group II were supplemented with CIMM premix at 1000 and 500 mg/kg, respectively. Specifically, the micro-mineral premix for the IMM group and that for CIMM group I provided the following mineral levels (mg/kg diet): Cu 7, Fe 200, Mn 30, Zn 70, I 1.6, Se 0.4, and Co 1.2. The micro-mineral premix for CIMM group II provided 50% of these mineral levels.

The dietary ingredients were crushed in a feed grinder (ZFJ-300, Jiangyin Ruizong Machinery Manufacturing Co., Ltd., Wuxi, China), passed through an 80 μm mesh, and then thoroughly mixed to formulate the experimental diets. Before feeding, the powdered diet was mixed with water (1.2 L/kg diet) to create a dough, which was then placed on a feeding table for the eels. The basal diet contained 45.86% crude protein and 6.34% crude lipid, with moisture, ash, total calcium, and total phosphorus levels of 6.56%, 12.12%, 2.77%, and 1.9%, respectively.

### 2.2. Fish and Feeding Trial

The American eels were raised in a recirculating aquaculture system with cement tanks (143 m^2^, 1.2 m height, and 0.9 m water depth) at a commercial farm (Jinjiangzhiman Aquatic Technology Co., Ltd., Zhangzhou, China). After the grading of eels, nine cement tanks with similar fish weight (about 2.33 g/fish and 41.30 kg/tank, equating to a stocking density of approximately 138 fish/m^3^) were randomly divided into three groups with three tanks (*n* = 3) per treatment. The eels were offered experimental diets to apparent satiation twice daily, at 5:00 a.m. and 5:00 p.m. Water temperature was maintained at 27–30 °C, while water quality parameters were as follows: pH 5.54 ± 0.43, dissolved oxygen 13.56 ± 0.59 mg/L, total ammonia nitrogen 10.17 ± 3.75 mg/L, and nitrite nitrogen levels 0.12 ± 0.05 mg/L. The trial lasted 56 days.

### 2.3. Sample Collection

All fish got 24 h fasting at the end of the experiment. Fifteen fish per tank were randomly selected and anesthetized with 50 mg/L eugenol. The intestines of the fish were aseptically removed on ice. A total of four foregut samples were designated for microbiome analysis, six foregut samples for metabolomic analysis, and three midgut samples for enzyme activity assays, respectively. These samples were frozen in liquid nitrogen and stored at −80 °C. Furthermore, two foregut samples were dissected, and their middle segments (approximately 2 cm) were preserved in Bouin’s solution for 24 h for intestinal tissue structure observation. Additionally, three fish per tank were randomly selected for body composition analysis.

### 2.4. Analytical Methods

#### 2.4.1. Growth Performance

At the end of the trial, all fish in each tank were weighed. The final fish weight (FFW) was calculated as the total weight of the fish in each cement tank at the end of the trial, added to the weight of the fish that died during the trial period. The weight gain rate (WGR), specific growth rate (SGR), feed intake (FI), feed efficiency (FE), protein efficiency rate (PER) and survival rate (SR) were calculated as follows:

WGR (%) = [final fish weight (kg/tank) − initial fish weight (kg/tank)]/initial fish weight (kg/tank) × 100;

SGR (%/d) = [ln(final fish weight (kg/tank)) − ln(initial fish weight (kg/tank))]/time period (day) × 100;

FI (%) = feed consumption (kg/tank)/(initial number of fish per tank − total number of dead fish per tank) × 100;

FE (%) = [final fish weight (kg/tank) − initial fish weight (kg/tank)]/feed intake (kg/tank) × 100;

PER (%) = 100 × [final fish weight (kg/tank) − initial fish weight (kg/tank)]/protein intake (kg/tank) × 100;

SR (%) = (initial number of fish per tank − total number of dead fish per tank)/initial number per tank × 100.

#### 2.4.2. Body Composition

Moisture content was determined by drying samples to a constant weight at 105 °C; crude protein was determined by the Kjeldahl method (FOSS 8400, Höganäs, Sweden); crude lipid was measured using the Soxhlet method (BUCHI 36880, Flawil, Switzerland); and crude ash content was assessed by combustion at 550 °C.

#### 2.4.3. Mineral Contents

Analysis of the calcium (Ca) and phosphorus (P) content in the whole-body was conducted using an ICP-OES prodigy spectrometer (Prodigy 7, Leeman Labs, Hudson, NH, USA). The contents of micro-minerals (Cu, Fe, Mn, and Zn) in the whole-body was determined using an atomic absorption spectrophotometer (TAS-990, Beijing Puxi, Beijing, China).

#### 2.4.4. Intestinal Digestive Enzymes and Antioxidant Capacity Determination

The three midgut samples from each group were removed from the −80 °C freezer and kept on ice to thaw. Approximately 0.5 g of intestinal tissue was taken from each sample and homogenized individually using a tissue homogenizer (Tissuelyser-24, Shanghai Jingxin Industrial Development Co., Ltd., Shanghai, China) with pre-cooled 0.86% saline added at a mass-to-volume ratio of 1:9 (g/mL). The homogenates were centrifuged for 10 min at 700× *g* at 4 °C. The supernatants from the three samples within the same group were pooled, mixed, and aliquoted into centrifuge tubes for subsequent use.

Protein concentrations were determined by the Lowry method [25]. Protease activity was assayed by the classic Folin-phenol method. The enzyme extract was pre-incubated at 40 °C for 2 min, mixed with pre-warmed 2% casein substrate (pH 7.2 phosphate buffer) at 1:1 volume ratio (*v*/*v*), and incubated at 40 °C for 10 min. The reaction was terminated with 0.4 mol/L trichloroacetic acid, and after centrifugation for 10 min at 960× *g*, the supernatant was collected. Tyrosine was quantified using 2-fold diluted Folin–Ciocalteu reagent and 0.4 mol/L sodium carbonate solution, with color development at 40 °C for 20 min and absorbance measured at 660 nm (a parallel blank control was included). Tyrosine concentration was determined via a standard curve (0–100 µg/mL), and one unit (U) of protease activity was defined as the amount of enzyme producing 1 μg tyrosine per minute per mg protein. The detection method of amylase is iodine-starch colorimetry, and that of lipase is turbidimetric assay. The detection method of superoxide dismutase (SOD) is colorimetry, that of catalase (CAT) is ammonium molybdate method, and that of malondialdehyde (MDA) is thiobarbituric acid method. All the above indicators were detected using commercial kits (Nanjing Jiancheng Biotechnic Institute, Nanjing, China).

#### 2.4.5. Histological Observation

Following fixation, the foregut samples were subjected to washing, dehydration, clarification, and paraffin embedding procedures. Serial 5 µm thick sections were cut and mounted on glass slides. These sections were then deparaffinized in xylene, rehydrated, stained with hematoxylin and eosin, and finally fixed with neutral balsam. After the stained sections were air-dried at room temperature, they were observed, photographed, and measured using an ortho-fluorescence microscope (BX80-JPA, Olympus, Tokyo, Japan). The villi length (VL) and muscular thickness (MT) were measured from the images using the ImageJ software (version 1.48; National Institutes of Health, Bethesda, MD, USA), with ten intact and well-oriented structures evaluated per section, and the mean value for each parameter from all biological replicates (tanks) was calculated per group.

#### 2.4.6. Intestinal Microbiota Profiling

The microbiota analysis was conducted on the IMM group and CIMM group I as the primary comparison. This approach was taken because the CIMM group I showed a significant improvement in growth, thereby allowing for a clearer investigation of the specific effects of CIMM on the intestinal microbiota. The intestinal microbiome analysis was performed on foregut samples. For this analysis, four foregut samples per tank were pooled to form two composite samples (by combining two samples into one). With three tanks per group, a total of six composite samples were analyzed for both the IMM and CIMM groups. Microbial community genomic DNA was extracted from intestinal samples of the American eel, with four samples collected from each group, using the E.Z.N.A.^®^ Soil DNA Kit (Omega Bio-tek, Norcross, GA, USA). DNA purity and quantification were determined through 1% agarose gel electrophoresis and a NanoDrop^®^ ND-2000 ultramicro spectrophotometer (Thermo Scientific Inc., Waltham, MA, USA). The V3-V4 hypervariable region of the 16S rRNA gene was amplified from the extracted DNA using universal primers (338 F: ACTCCTACGGGAGGCAGCAG and 806 R: GGACTACHVGGGTWTCTAAT) via a polymerase chain reaction (PCR) using the ABI GeneAmp^®^ 9700 PCR thermocycler (ABI, Foster City, CA, USA). The resulting PCR products were purified, and equimolar amounts from each sample were pooled to construct the sequencing libraries. Next-generation sequencing analysis of the 16S rRNA genes was performed on the Illumina MiSeq PE300 platform (Illumina, San Diego, CA, USA). The raw sequencing data were processed as follows: quality filtering and assembly of paired-end reads were performed using fastp and FLASH, respectively. The high-quality sequences were then clustered into operational taxonomic units (OTUs) at a 97% similarity threshold using the UPARSE algorithm. Taxonomic classification of the OTU representative sequences was conducted using the RDP Classifier against the Silva (v138) 16S rRNA database with a confidence threshold of 0.7. Based on the OTU data, alpha diversity indices were calculated using Mothur v1.30.1. Differences in alpha diversity indices among groups were assessed by the Wilcoxon rank-sum test, with *p* < 0.05 considered statistically significant. Principal component analysis (PCA) was performed based on the Bray–Curtis dissimilarity matrix to visualize the overall structural differences in microbial communities between the two groups. The statistical significance of the group separation was assessed using Permutational Multivariate Analysis of Variance (PERMANOVA) with 999 permutations. The relative abundance of bacterial communities at different taxonomic levels (phylum to genus) was calculated and visualized to describe the overall structure of the microbial assemblages.

#### 2.4.7. The Intestinal Metabolome Analysis

As with the microbiome analysis, the metabolome profiling was performed exclusively on foregut samples from the IMM group and CIMM group I. For this analysis, six foregut samples per tank were pooled to form two composite samples (by combining three samples into one). With three tanks per group, a total of six composite samples were analyzed for both the IMM and CIMM groups. Metabolites were extracted using a 400 μL solution of methanol and water (4:1, *v*/*v*), followed by extraction according to the protocol provided by Majorbio Bio-Pharm Technology Co., Ltd. (Shanghai, China) for non-targeted metabolite profiling. A 10 μL portion of the extracted samples was analyzed using an ultra-high performance liquid chromatography (UHPLC) system coupled with a quadrupole-time-of-flight mass spectrometer (Triple TOFTM 5600+, AB SCIEX, Framingham, MA, USA) for LC-MS analysis. Raw data from UPLC-MS/MS were processed using Progenesis QI software (version 2.4, Waters Corporation, Milford, MA, USA) for peak picking, alignment, and metabolite identification by querying the HMDB, Metlin, and self-compiled Majorbio Database (MJDB). The resulting data matrix was further analyzed on the Majorbio Cloud Platform (https://cloud.majorbio.com). Statistical significance of metabolites was determined using a combination of multivariate and univariate analyses. Specifically, metabolites with a Variable Importance in Projection (VIP) > 1.0 from an orthogonal partial least squares-discriminant analysis (OPLS-DA) model and a *p*-value < 0.05 from a Student’s *t*-test were defined as significantly differential metabolites. These significantly differential metabolites were then subjected to metabolic pathway enrichment analysis based on the KEGG database. The *p*-values from the enrichment analysis were adjusted for multiple testing using the Benjamini–Hochberg (BH) procedure to control the false discovery rate (FDR). Pathways with an adjusted *p*-value (FDR) < 0.05 were considered statistically significantly altered.

### 2.5. Integrated Analysis of Microbiota and Metabolome

To investigate microbiota-metabolite associations, Spearman correlation analysis was performed between the top 10 most abundant genera and differential metabolites from significantly altered pathways. Technical replicates were integrated at the tank level by averaging genus abundances and metabolite concentrations from composite samples. Specifically, for each tank, the two composite samples (for microbiota and metabolomics, respectively) were first averaged to generate a single value per tank for each bacterial genus and metabolite. These tank-level averages were then used in the subsequent Spearman correlation analysis between microbiota and metabolome. Correlations with |r| > 0.8 and *p* < 0.05 were considered significant.

### 2.6. Statistical Analysis

The results are presented as means ± SD. Statistical analysis was conducted using SPSS 22.0 software (SPSS, Chicago, IL, USA). Prior to ANOVA, the assumptions of normality (Shapiro–Wilk test) and homogeneity of variances (Levene’s test) were evaluated and met. Then, one-way ANOVA analysis followed by Duncan’s test was employed to compare means between treatments at a significance level of *p* < 0.05. Correlation coefficient (Spearman) was analyzed using R (version 4.1.2).

### 2.7. AI Tool Disclosure

During the preparation of this work, the authors used DeepSeek, V3.2, a generative AI model developed by Hangzhou Deepseek Artificial Intelligence Basic Technology Research Co., Ltd. (Hangzhou, China), exclusively for language polishing, grammar checking, and improving the readability of the text. The authors carefully reviewed, edited, and validated all AI-generated suggestions to ensure alignment with the original scientific intent and accuracy. All scientific content, data, interpretations, and conclusions are solely the responsibility of the authors.

## 3. Results

### 3.1. Growth Performance

Table 2 presents the effects of CIMM on the growth performance of juvenile American eels. Compared with the IMM group, the WGR, SGR, FI, FE, and PER of the CIMM group I were significantly increased (*p* < 0.05). CIMM group II also had a significantly higher FI than that of the IMM group (*p* < 0.05). For all other growth performance parameters, CIMM group II showed no significant differences from those of the other groups (*p* > 0.05).

### 3.2. The Content and Retention Rate of Minerals in the Whole-Body

**Figure 1:**
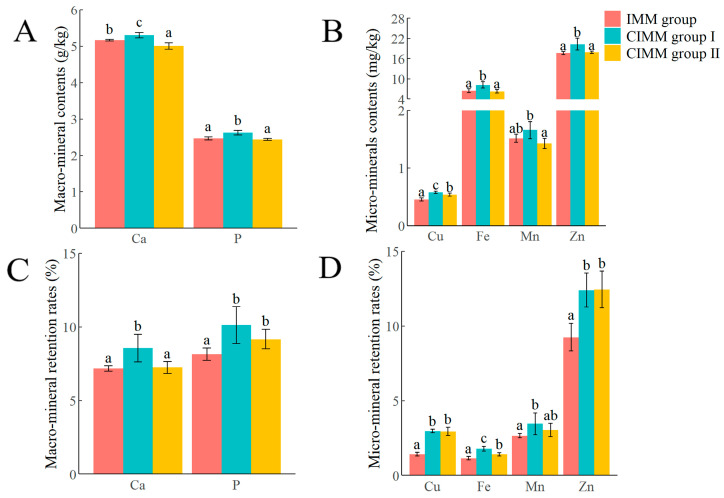
The effects of CIMM on whole-body mineral content and retention rates in juvenile American eels (wet sample). (**A**) Calcium (Ca) and phosphorus (P) contents. (**B**) Copper (Cu), iron (Fe), manganese (Mn), and zinc (Zn) contents. (**C**) The retention rates of Ca and P. (**D**) The retention rates of Cu, Fe, Mn, and Zn. Letters (a–c) above bars indicate significant differences (*p* < 0.05). Data are presented as mean ± SD (*n* = 3 tanks).

Analysis of whole-body mineral content (Figure 1A,B) revealed significantly higher levels of Ca, P, Cu, Fe, Mn, and Zn in the CIMM group I compared to the CIMM group II and the IMM group (*p* < 0.05). In addition, compared with the IMM group, the CIMM group II had a significantly higher mineral content of Cu and a significantly lower mineral content of Ca (*p* < 0.05), while there were no significant differences in the mineral contents of P, Fe, Zn and Mn (*p* > 0.05).

The mineral retention rates in the whole-body of juvenile American eels, as shown in Figure 1C,D, indicated that the retention rates of Ca, P, Cu, Fe, Mn, and Zn were significantly higher in the CIMM group I compared to the IMM group (*p* < 0.05). Additionally, while the retention rates of Ca and Fe were significantly higher in the CIMM group I than in the CIMM group II, no significant differences were found in the retention rates of other minerals between the two groups (*p* > 0.05). Furthermore, the retention rates of P, Cu, Fe, and Zn were significantly higher in the CIMM group II than in the IMM group (*p* < 0.05). No significant differences were observed in the retention rates of Ca and Mn between the IMM group and the CIMM group II (*p* > 0.05).

### 3.3. The Whole-Body Composition

The effect of CIMM on the whole-body composition of juvenile American eels is presented in Table 3. There were no significant differences in the levels of moisture, ash, crude protein, and crude lipid among different treatment groups (*p* > 0.05).

### 3.4. The Activity of Digestive Enzymes in the Intestine

Table 4 displays the effects of CIMM on the activity of intestinal digestive enzymes. Compared with the IMM group, the activity of amylase and lipase in the intestine of the CIMM group I increased significantly (*p* < 0.05). However, there were no significant differences in the activity of these enzymes between the CIMM group I and the CIMM group II (*p* > 0.05). Additionally, there were no significant differences in protease activity among the IMM group, CIMM group I, and CIMM group II (*p* > 0.05).

### 3.5. The Intestinal Antioxidant Capacity

The effects of CIMM on the intestinal antioxidant capacity of juvenile American eels are presented in Table 5. The CIMM group I exhibited significantly increased activities of SOD and CAT in comparison to the IMM group (*p* < 0.05). Conversely, the MDA level was significantly lower in the CIMM group I than that in the IMM group (*p* < 0.05). In the CIMM group II, SOD enzyme activity was also significantly higher than that observed in the IMM group (*p* < 0.05); however, the CAT enzyme activity and the MDA level did not exhibit significant differences when compared to the IMM group (*p* > 0.05).

### 3.6. Intestinal Morphology

**Figure 2:**
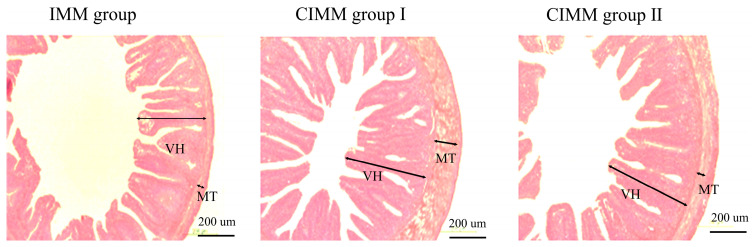
Effects of CIMM on the intestinal morphology parameters of juvenile American eels (*n* = 3 tanks).

As shown in Table 6 and Figure 2, the VL of the intestine in the IMM group was significantly lower than that in the CIMM group I and CIMM group II (*p* < 0.05), and there was no significant difference in the VL between the CIMM group I and the CIMM group II (*p* > 0.05). The MT of the intestine in the CIMM group was significantly higher than that in the IMM group and the CIMM group II (*p* < 0.05), while the CIMM group II had a significantly higher MT than that in the IMM group (*p* < 0.05).

### 3.7. Composition and Diversity of Intestinal Microbiota

**Figure 3:**
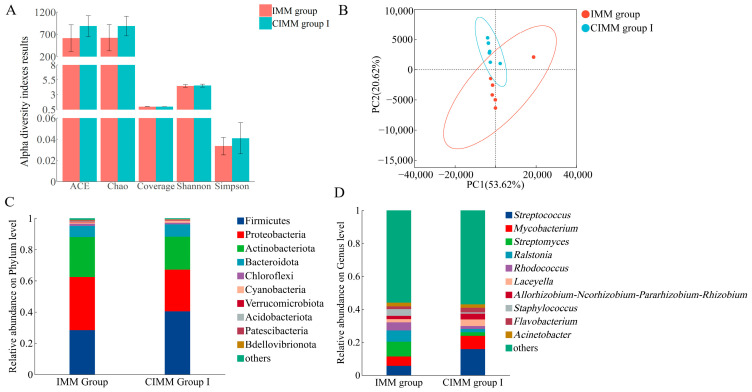
Effects of CIMM on the composition and diversity of intestinal microbiota of juvenile American eels. (**A**) Alpha diversity indexes results. Data are presented as mean ± SD. (**B**) PCA results. (**C**) Relative abundance of species at the phylum level of the CIMM group I and the IMM group. (**D**) Relative abundance of species at the genus level of the CIMM group I and the IMM group. Microbiome analyses are based on *n* = 6 composite samples per group (2 composite samples per tank × 3 replicate tanks). Statistical tests were performed using the composite sample as the analytical unit.

The effects of CIMM on the composition and diversity of intestinal microbiota of juvenile American eels are shown in Figure 3. No significant differences were observed in the Abundance-based Coverage Estimator (ACE), Chao1 diversity indexes, Shannon and Simpson diversity indexes between the IMM and CIMM group I (*p* > 0.05) (Figure 3A). Principal component analysis (PCA) revealed a clear separation in microbial community structure between the CIMM group I and the IMM group (Figure 3B), supporting a distinct difference between the two treatments. Specifically, principal component 1 (PC1, horizontal axis) explained 53.62% of the variation, and principal component 2 (PC2, vertical axis) explained 20.62%, cumulatively accounting for 74.24% of the total microbial community variation. As shown in Figure 3C,D, the microbial abundance differed between the IMM group and CIMM group I at the phylum and genus levels, respectively. At the phylum level, Firmicutes and Proteobacteria were detected as the dominant phyla in juvenile American eel intestine (Figure 3C). At the genus level, *Streptococcus*, *Mycobacterium*, *Streptomyces*, *Ralstonia*, *Rhodococcus* and *Laceyella* were the dominant genera in juvenile American eel intestine (Figure 3D).

### 3.8. Untargeted Metabolomics Profiling of the Intestine

**Figure 4:**
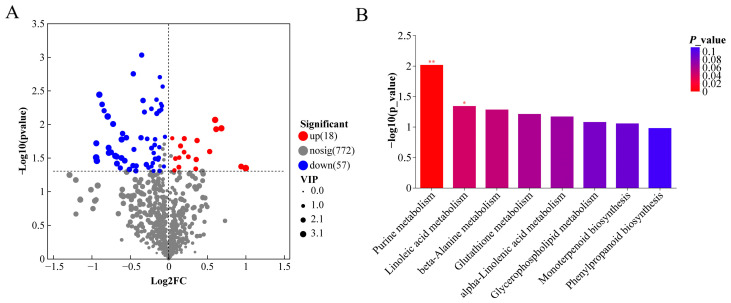
Comparative analysis of the intestinal metabolome between CIMM group I and IMM group juvenile American eels. (**A**) Volcano plots of differential metabolites. Red represents the up-regulation; blue represents the down-regulation; gray represents no significant change. (**B**) KEGG enrichment analysis of the differential metabolites in juvenile American eels of the CIMM group I compared to the IMM group. * represents the significant level, ** represents the extremely significant level. Statistical identification of differential metabolites and pathway enrichment are based on *n* = 6 composite samples per group (2 composite samples per tank × 3 replicate tanks). Statistical tests were performed using the composite sample as the analytical unit.

As illustrated in the volcano plots (Figure 4A), comparative analysis between the CIMM group I and the IMM group identified 75 differential metabolites. Of these, 18 were significantly increased (*p* < 0.05), and 57 were significantly decreased in the CIMM group I (*p* < 0.05). Additionally, the primary metabolic pathways associated with the different metabolites were summarized in Figure 4B. Among these pathways, purine metabolism, linoleic acid metabolism, beta-Alanine metabolism, Inflammatory mediator regulation of TRP channels and Glutathione metabolism were the primary altered metabolic pathway. Purine metabolism and linoleic acid metabolism were found to be statistically significant (*p* < 0.05). The associated differential metabolites for these pathways were presented in Table 7. The levels of hypoxanthine, deoxyinosine, 9,10,13-trihydroxyoctadecadienoic acid (9,10,13-TriHOME) and spermidine in the CIMM group I were significantly lower than those in the IMM group.

### 3.9. Correlation Between Intestinal Microbiota and Differential Metabolites

**Figure 5:**
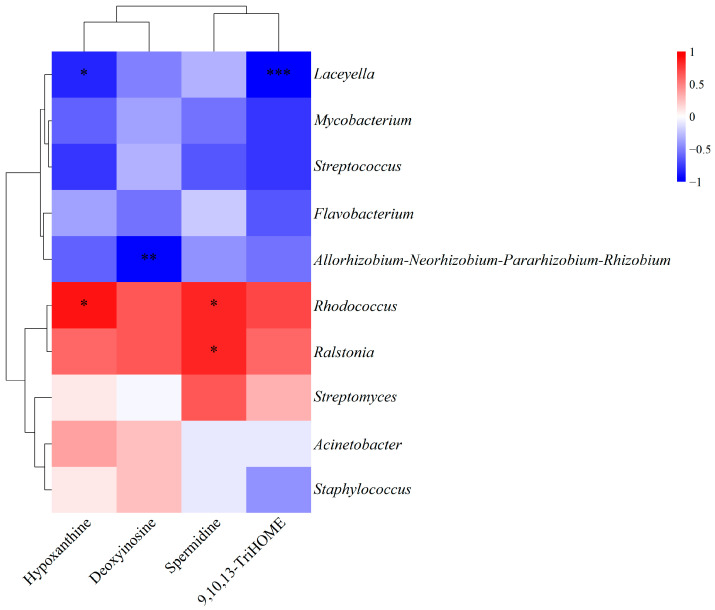
Correlation analysis between key genera and metabolites. * *p* < 0.05, ** *p* < 0.01, *** *p* < 0.001. The heatmap shows Spearman’s correlation coefficients; statistical analysis was performed using the tank as the experimental unit (*n* = 3 tanks).

To further explore potential functional links, we performed a Spearman correlation analysis between specific bacterial genera and metabolites (Figure 5). *Laceyella* abundance was inversely correlated with 9,10,13-TriHOME (*p* < 0.001) and hypoxanthine levels (*p* < 0.05). *Rhodococcus* exhibited positive correlations with both spermidine and hypoxanthine (*p* < 0.05). A significant positive correlation was observed between *Ralstonia* and spermidine (*p* < 0.05). Additionally, the *Allorhizobium-Neorhizobium-Pararhizobium-Rhizobium* showed a significant negative correlation with deoxyinosine (*p* < 0.01).

## 4. Discussion

### 4.1. Growth Performance

The experimental findings demonstrated that under commercial RAS conditions, the addition of equal amounts of CIMM instead of IMM in the feed significantly improved the growth performance of American eels (WGR, SGR, FI, FE, and PER). The improvement might be attributed to its higher bioavailability and its protective coating that effectively prevented micro-minerals from damaging nutrients. This confirmed and extended the positive conclusions drawn from prior controlled laboratory studies [9]. It demonstrates that the effectiveness of CIMM observed in the lab is maintained in a commercial RAS.

However, a key difference was observed: the CIMM group II (with 50% reduced CIMM supplementation) did not show significant differences in other growth performance indicators compared to the IMM group, except for feed intake. This contrasted with previous laboratory findings where the same reduction in CIMM supplementation still showed significant growth benefits [9]. This discrepancy may be related to several factors. First, the stocking density was higher in the commercial trial (138 fish/m^3^) than in the laboratory study (94 fish/m^3^) [9], and elevated stocking density could increase nutritional requirements in fish [26]. Second, chronic stress is inherent to commercial systems [27]. Third, early-stage juvenile fish have higher metabolic demands [28]; the fish in this study had a lower initial body weight (2.33 g/fish) compared to those in the laboratory study (12.05 g/fish) [9].

### 4.2. Mineral Contents, Mineral Retention Rate, and Proximate Body Composition in the Whole-Body

This study demonstrates that dietary CIMM are more effectively utilized and retained than traditional IMM in juvenile American eels. This conclusion is supported by the significantly higher whole-body retention rates of Ca, P, Cu, Fe, Mn, and Zn in the CIMM group I compared to the IMM group under commercial culture conditions. The significantly higher whole-body content of these minerals in the CIMM group I further corroborates this enhanced utilization. While the superiority of CIMM in enhancing mineral retention was previously documented in a controlled laboratory environment [9], the present study confirms that this benefit is reproducible in a commercial farm setting, strengthening the evidence for its application.

The enhanced mineral utilization is further underscored by the performance of the CIMM group II. Despite receiving only 50% of the mineral supplementation, this group still exhibited significantly higher whole-body retention rates of P, Cu, Fe, and Zn than the IMM group. The likely reason for this overall improvement is the protective carbohydrate matrix of CIMM, which isolates the minerals in the digestive tract. This coating is expected to reduce the formation of insoluble complexes between the minerals and dietary antagonists such as phytates and oxalates, a known cause of the low absorption and utilization efficiency of IMM [11,17]. Together, these results indicate that the coating technology not only enhances mineral retention but can maintain this efficacy even at a substantially reduced supplementation level.

Proximate composition is an indicator for assessing the overall nutritional and physiological condition of a fish [29]. The results obtained under laboratory conditions indicated that CIMM did not alter the body composition of juvenile American eels [9]. Consistently, this experiment found that in the commercial application, the addition of CIMM to the feed did not result in significant differences in moisture, ash, crude protein, or crude lipid content in juvenile American eels. This stability in body composition, despite the clear improvements in growth performance, indicates that the growth advantage from CIMM was not achieved by altering the fish’s body composition. Instead, these results suggest that the benefits of CIMM stem from an improved supply of micro-minerals. This improved supply, as reflected in the markedly increased mineral retention, allows these micro-minerals to fulfill their fundamental roles as essential cofactors for enzymes involved in digestion and antioxidant defense, as will be detailed in the following sections.

### 4.3. The Activity of Digestive Enzymes in the Intestine

The activity of intestinal digestive enzymes serves as a vital indicator of fish’s ability to digest and absorb feed, reflecting their efficiency in nutrient utilization [30]. Studies have confirmed that minerals are essential for the regulation of digestive enzyme activities [1]. Previous research revealed that the activities of amylase and lipase in the intestines of hybrid tilapia *Oreochromis niloticus* (L.) × *Oreochromis aureus* (*Steindachner*) increased with the supplementation of Cu, Fe, or Zn, while the activity of proteases showed no changes [31]. Similarly, our results demonstrated that, compared with the IMM group, the activities of amylase and lipase in the intestines of the CIMM group I increased significantly. This conclusion is consistent with the experimental results in Yin’s research on juvenile American eels conducted under laboratory conditions [9]. This enhancement could be linked to the high utilization efficiency of CIMM. The improved bioavailability of key minerals such as zinc led to improved intestinal morphology and increased epithelial cell count, thereby indirectly promoting the secretion of digestive enzymes [32,33]. Considering the previously mentioned growth performance results, the feed utilization efficiency in the CIMM group I was significantly higher than that in the IMM group. This suggests that dietary supplementation with CIMM in juvenile American eels promotes the digestion and absorption of nutrients by enhancing digestive enzyme activity, thereby improving feed conversion rates and ultimately leading to enhanced growth performance. However, only lipase activity was significantly elevated in the CIMM group II compared to the IMM group. This result contrasts with the laboratory findings, where the same reduction in CIMM supplementation significantly enhanced both amylase and lipase activities [9]. It indicates that a 50% supplementation level of CIMM in a commercial RAS is insufficient to achieve the efficacy observed under controlled laboratory conditions.

### 4.4. The Intestinal Antioxidant Capacity

Intestinal antioxidant capacity is important for maintaining intestinal health [34]. The antioxidant system is made up of various antioxidant enzymes, such as SOD and CAT, which effectively neutralize oxygen radicals and help prevent oxidative stress [35]. The levels of MDA in the intestine serve as indicators of the extent of damage inflicted by reactive oxygen free radicals [36]. Micro-minerals, as integral components of antioxidant enzymes, play a crucial role in ensuring the proper functioning of these enzymes [37]. Research has found that the addition of an appropriate amount of Fe can significantly increase the activities of SOD and CAT in the intestines of yellow catfish (*Pelteobagrus fulvidraco*), while significantly reducing the levels of MDA in the intestine [38]. Similar findings have been reported in other fish species, including Nile tilapia (*Oreochromis niloticus*) [39], blunt snout bream (*Megalobrama amblycephala*) [40], and Jian carp (*Cyprinus carpio* var. Jian) [41]. Consistent with these previous studies, our findings revealed that, compared to the IMM group, the CIMM group I showed a significant increase in the activities of intestinal SOD and CAT, while the levels of intestinal MDA in the CIMM group I were significantly lower than those in the IMM group. These findings indicate the importance of CIMM in promoting antioxidant activity and intestinal health in juvenile American eels both under laboratory conditions [9] and in commercial RAS. However, in the CIMM group II, only the intestinal SOD enzyme activity was significantly higher than that in the IMM group. This suggests that the reduced amount of CIMM has a limited effect on enhancing the antioxidant capacity of the intestines in juvenile American eels.

### 4.5. Intestinal Morphology

Intestinal morphology is a major indicator of intestinal health [42]. In fish, the intestine serves as the primary organ for nutrient absorption [43]. The intestines contract through the muscular layer, promoting the complete mixing of food with digestive juices, thereby increasing the speed of nutrient digestion [44]. The increase in the height of intestinal villi signifies a greater absorptive surface area, thereby improving the nutritional absorption capacity of fish [45]. In this study, compared to the IMM group, the CIMM groups resulted in a significant increase in the height of intestinal villi and the thickness of the intestinal muscular layer in juvenile American eels. These findings suggested that the inclusion of CIMM in the diet is advantageous for enhancing the absorption function of the intestines in juvenile American eels. Similarly, it was discovered that CIMM can significantly increase the height of intestinal villi and the thickness of the intestinal muscular layer in juvenile American eels conducted under laboratory conditions [9]. This indicates that dietary CIMM supplementation can preserve intestinal morphological integrity and promote intestinal health in juvenile American eels reared under both commercial RAS and controlled laboratory conditions. The beneficial effect of CIMM on intestinal morphology has not been reported in other fish species besides American eels, whereas it has been observed in terrestrial livestock such as growing sheep [21], broilers [18], laying hens [46].

### 4.6. Intestinal Microbial Diversity and Relative Abundance

The intestinal microbiota plays a crucial role in maintaining host intestinal health [47,48]. This study employed intestinal microbiome sequencing to compare the effects of CIMM versus traditional IMM on the intestinal microbiota of juvenile American eels under commercial culture conditions. The ACE and Chao1 indices serve as estimators of community richness [49,50], while the Shannon and Simpson indices reflect community diversity, incorporating both richness and evenness [51]. In this study, no significant differences were observed in any of these parameters between the CIMM group I and the IMM group, indicating that the addition of CIMM to the feed did not significantly alter the overall structure of the intestinal microbiota.

Firmicutes and Proteobacteria have been found as the predominant bacterial phyla in the intestine of juvenile American eels, consistent with findings in other freshwater fish species such as largemouth bass (*Micropterus salmoides*) [52,53], yellow catfish (*Pelteobagrus fulvidraco*) [54], and gibel carp (*Carassius auratus gibelio*) [55]. Compared to the IMM group, the CIMM group I exhibited an increased relative abundance of *Firmicutes* and a reduced relative abundance of Proteobacteria. Firmicutes are characterized by their significant contribution to the production of short-chain fatty acids in the intestine [56]. In contrast, Proteobacteria is a phylum that encompasses a wide range of potentially pathogenic genera [57]. A similar microbial shift, specifically an increase in Firmicutes and a decrease in Proteobacteria, has been reported in swamp eels following copper exposure, though it was associated with enteritis [58]. In the present study, the same phylum-level change co-occurred with healthy intestinal morphology. The appropriate levels of micro-minerals can promote the growth of beneficial bacteria, thereby enhancing overall intestinal health [1,52]. The change in dominant phyla within the intestinal microbiota may be related to CIMM providing more suitable micro-mineral levels for juvenile American eels.

### 4.7. Intestinal KEGG Pathways and Differential Metabolites

The metabolic levels in the intestinal contents were assessed and analyzed to further understand the impact of CIMM on the intestinal health of juvenile American eels. Purine metabolism plays a critical role in the response to oxidative stress [59], and purine catabolism helps protect the organism from oxidative damage [60]. Hypoxanthine, an intermediate in purine metabolism [61], is often used as a marker of oxidative stress when its levels are elevated [62,63,64]. In this study, the levels of hypoxanthine in the intestines of the CIMM group I were significantly lower than those in the IMM group. This suggests that the addition of traditional IMM in the feed, compared to CIMM, may be associated with oxidative stress in the intestines of juvenile American eels. Adenosine deaminase catalyzes the deamination of deoxyadenosine into deoxyinosine [65]. In this experiment, the content of intestinal deoxyinosine the CIMM group I was significantly decreased, indicating that the intestinal purine metabolic decomposition in the CIMM group I might be enhanced. 9,10,13-TriHOME is an end product of linoleic acid oxidation and is associated with oxidative stress [66]. Surendran et al. [67] demonstrated that oxidative stress results in a significant increase in 9,10,13-TriHOME levels in human low-density lipoprotein, indicating that 9,10,13-TriHOME may serve as an additional biomarker for identifying oxidative stress. In the present study, the CIMM group I exhibited decreased levels of 9,10,13-TriHOME, which might be related to the reduction in oxidative stress in the intestine. In this experimental study, the beta-Alanine metabolism and Glutathione metabolism pathways were both enriched in the differential metabolite spermidine. Spermidine has been shown to play a role in the regulation of inflammatory responses, with its concentrations significantly increasing at the sites of inflammation in infections and injuries [68]. Overall, the metabolomic results indicated that, compared to CIMM, traditional IMM may be associated with higher oxidative stress in the intestines of juvenile American eels, which is consistent with the results of the intestinal antioxidant capacity. The reason could be related to the involvement of micro-minerals in the composition of various antioxidant enzymes. For instance, Cu, Zn, and Mn are components of SOD, Fe serves as a cofactor for CAT, and Se is a component of GSH-Px.

### 4.8. Correlation Between Intestinal Microbiota and Differential Metabolites

Spearman correlation analysis was performed to explore potential associations between the top 10 bacterial genera and the differential metabolites. Several significant correlations were identified, offering insights into the interplay between the microbiota and metabolome in the intestine of juvenile American eels.

The abundance of *Laceyella* was inversely correlated with the levels of 9,10,13-TriHOME and hypoxanthine. Given that *Laceyella* is a thermophilic actinobacterium commonly found in high-temperature compost [69] and the experimental diet contained extruded soybean, it is plausible that this bacterium originated from the feed. Consequently, the higher abundance of *Laceyella* in the CIMM group I may reflect increased feed intake associated with improved intestinal health, rather than a direct probiotic effect. Further studies are needed to clarify whether *Laceyella* can serve as an indicator of feed intake and to understand its ecological role in the intestinal environment.

The positive correlation observed between the opportunistic pathogens *Rhodococcus* and *Ralstonia* with spermidine is noteworthy [70,71]. Considering that *Ralstonia* is known to promote inflammation in human and spermidine levels typically increase during inflammatory responses [68,70], their co-occurrence may be associated with aggravated intestinal inflammation. The concurrent reduction in both these bacteria and spermidine in the CIMM group I suggests that replacing IMM with CIMM could be related to improved intestinal inflammatory status. Although the pro-inflammatory role of these bacteria in fish remains to be confirmed, the changes in both the microbiota and metabolome are consistent with a beneficial role of CIMM in maintaining intestinal homeostasis.

It should be noted that, due to the small body size of juvenile eels, intestinal samples were pooled within each tank to obtain sufficient material for microbial and metabolomic profiling. Therefore, the obtained data reflect responses to dietary treatment rather than inter-individual variation among fish. The Spearman correlations between microbiota and metabolites are exploratory and hypothesis-generating. Future studies employing individual sampling designs with larger sample sizes are needed to elucidate individual host responses and validate these potential interactions.

Compared to the preceding laboratory study [9], the higher ammonia nitrogen (10.17 ± 3.75 mg/L) and lower pH (5.54 ± 0.43) conditions in this commercial RAS may have exerted a potential influence on the micro-mineral requirements of juvenile American eels. Elevated environmental ammonia is a known inducer of oxidative stress, which would increase the demand for antioxidant micronutrients (e.g., Cu, Zn, Mn, Se) that serve as cofactors for enzymes such as SOD and GSH-Px [4,72]. In addition, low pH may increase the solubility of micro-mineral in feed [73], whereas in a low-pH environment, H^+^ may inhibit the absorption of minerals in fish by competing with mineral ions for transporter sites, disrupting intestinal ion balance, or interfering with the binding between mineral ions and mucins [74]. The 500 mg/kg CIMM supplementation, though effective under optimized laboratory conditions [9], appeared insufficient in this commercial environment may be associated with this environmental stress.

Taken together, under commercial RAS conditions in the present study, juvenile American eels fed the 500 mg/kg diet exhibited significantly lower intestinal CAT activity and muscular thickness, alongside numerically lower WGR and SGR, compared to those fed 1000 mg/kg CIMM. In contrast, under laboratory conditions, juvenile American eels fed a diet with 500 mg/kg CIMM showed no significant differences in intestinal CAT activity, muscular thickness, WGR, or SGR compared to those fed 1000 mg/kg CIMM, although WGR and SGR values were slightly higher at the lower level [9]. Additionally, while the 500 mg/kg CIMM diet produced significantly higher WGR and SGR than the IMM-supplemented diet in the laboratory, this advantage was only numerical and non-significant on the farm. These results collectively indicate that the requirement in commercial systems could be higher than that under controlled laboratory conditions.

## 5. Conclusions

Under the specific mineral composition and environmental conditions tested, this study demonstrates the superior efficacy of the CIMM over the IMM for juvenile American eels in commercial aquaculture. Furthermore, integrated multi-omics analysis revealed that CIMM supplementation beneficially regulated the intestinal microbiota and reduced metabolites linked to oxidative stress and inflammation, thereby providing mechanistic insights at the multi-omics level.

Notably, a 50% reduction in CIMM supplementation level, which showed significant growth promotion compared to IMM in a controlled laboratory study, shows only a non-significant trend toward growth promotion in a commercial RAS. This result indicates that, for juvenile American eels under commercial RAS conditions, this 50% reduction in CIMM supplementation level is insufficient for promoting growth. These results imply that the dietary requirement for micro-minerals may be higher in commercial RAS than in laboratory settings. In summary, for commercial RAS feed formulation of juvenile American eels, it is recommended to replace IMM with CIMM at the validated level of 1000 mg/kg, while the optimum supplementation level warrants further investigation.

## Figures and Tables

**Table 1:** Ingredients and proportion of experimental diets (%, dry weight basis).

Ingredients	IMM Group	CIMM Group I	CIMM Group II
White fish meal	28.00	28.00	28.00
Brown fish meal	40.00	40.00	40.00
α-starch	24.00	24.00	24.05
Yeast powder	4.00	4.00	4.00
Extruded soybean	2.00	2.00	2.00
Monocalcium phosphate	0.50	0.50	0.50
Choline chloride	0.50	0.50	0.50
Magnesium sulfate heptahydrate	0.50	0.50	0.50
Vitamin premix ^a^	0.40	0.40	0.40
Mineral premix ^b^	0.10	0.10	0.05
Total	100	100	100

^a^ For detailed composition, see Supplementary Table S1. ^b^ For detailed compositions of the inorganic (IMM) and coated (CIMM I and II) micro-mineral premixes, see Supplementary Table S2.

**Table 2:** Effects of CIMM on the growth performance of juvenile American eels.

Items	IMM Group	CIMM Group I	CIMM Group II
IFW (kg/tank)	41.30 ± 1.61	41.40 ± 1.40	41.20 ± 1.08
FFW (kg/tank)	85.50 ± 2.01 ^a^	98.73 ± 3.03 ^b^	91.87 ± 3.92 ^ab^
WGR (%)	107.08 ± 1.92 ^a^	140.43 ± 13.89 ^b^	122.88 ± 10.01 ^ab^
SGR (%/d)	1.30 ± 0.02 ^a^	1.55 ± 0.12 ^b^	1.43 ± 0.08 ^ab^
FI (kg/tank)	68.81 ± 1.34 ^a^	79.03 ± 3.07 ^b^	74.82 ± 2.26 ^b^
FE (%)	64.23 ± 0.34 ^a^	72.46 ± 2.81 ^b^	67.60 ± 3.58 ^ab^
PER (%)	141.10 ± 0.75 ^a^	158.00 ± 6.14 ^b^	147.87 ± 7.82 ^ab^
SR (%)	99.03 ± 0.01	99.87 ± 0.02	98.84 ± 0.03

Values with the different letters in the same line are significantly different at *p* < 0.05. IFW, Initial fish weight; FFW, Final fish weight; WGR, Weight gain rate; SGR, Specific growth rate; FI, Feed intake; FE, Feed efficiency; PER, Protein efficiency rate; SR, Survival rate. Data are presented as mean ± SD (*n* = 3 tanks).

**Table 3:** Effect of CIMM on the whole-body composition of juvenile American eels (%).

Items	IMM Group	CIMM Group I	CIMM Group II
Moisture	74.01 ± 0.01	74.16 ± 0.48	74.51 ± 0.14
Ash	1.78 ± 0.04	1.79 ± 0.06	1.78 ± 0.04
Crude protein	15.34 ± 0.51	15.29 ± 0.18	15.07 ± 0.09
Crude lipid	8.94 ± 0.28	9.26 ± 0.2	8.99 ± 0.29

Data are presented as mean ± SD (*n* = 3 tanks).

**Table 4:** Effects of CIMM on the activity of intestinal digestive enzymes of juvenile American eels.

Items	IMM Group	CIMM Group I	CIMM Group II
Amylase (U/mg prot)	0.37 ± 0.04 ^a^	0.45 ± 0.01 ^b^	0.42 ± 0.03 ^ab^
Lipase (U/g prot)	19.37 ± 0.39 ^a^	28.98 ± 2.98 ^b^	25.09 ± 0.22 ^b^
Protease (U/mg prot)	58.34 ± 4.06	63.33 ± 1.51	60.93 ± 3.76

Values with the different letters in the same line are significantly different at *p* < 0.05. Data are presented as mean ± SD (*n* = 3 tanks).

**Table 5:** Effects of CIMM on the intestinal antioxidant capacity of juvenile American eels.

Items	IMM Group	CIMM Group I	CIMM Group II
SOD (U/mg prot)	59.86 ± 4.03 ^a^	79.72 ± 2.38 ^b^	76.61 ± 2.88 ^b^
CAT (U/mg prot)	7.11 ± 0.69 ^a^	8.55 ± 0.31 ^b^	7.21 ± 0.69 ^a^
MDA (nmol/mg prot)	34.69 ± 2.24 ^b^	28.33 ± 0.48 ^a^	31.13 ± 1.93 ^ab^

Values with the different letters in the same line are significantly different at *p* < 0.05. Data are presented as mean ± SD (*n* = 3 tanks).

**Table 6:** Effects of CIMM on the intestinal morphology of juvenile American eels.

Items	IMM Group	CIMM Group I	CIMM Group II
VL (µm)	352.56 ± 25.54 ^a^	443.82 ± 18.73 ^b^	405.57 ± 50.35 ^b^
MT (µm)	65.27 ± 4.05 ^a^	140.07 ± 22.09 ^c^	95.89 ± 4.18 ^b^

Values with the different letters in the same line are significantly different at *p* < 0.05. VL, Villi length; MT, Muscular thickness. Data are presented as mean ± SD (*n* = 3 tanks).

**Table 7:** Differential metabolites in the enriched metabolic pathways.

Pathway	Differential Metabolites	*p*-Value
Purine metabolism	Hypoxanthine ↓; Deoxyinosine ↓	0.0097
Linoleic acid metabolism	9,10,13-TriHOME ↓	0.0458
beta-Alanine metabolism	Spermidine ↓	0.0521
Glutathione metabolism	Spermidine ↓	0.0616

The *p*-values in the table correspond to the enrichment significance of the KEGG pathways to which the differential metabolites belong. Down arrows (↓) indicate metabolites significantly downregulated (*n* = 6 composite samples per group).

## Data Availability

The data that support the findings of this study are available from the corresponding author upon reasonable request.

## Supplementary Materials

The following supporting information can be downloaded at: https://www.mdpi.com/article/10.3390/ani16020324/s1, Table S1. Composition of the vitamin premix. Table S2. Composition of the Micro-mineral premixes (mg per kg of diet). File S1. Differential metabolite.
