# Supplementary material for: Effect of Coated Inorganic Micro-Minerals on Growth, Mineral Retention, and Intestinal Health in Juvenile American Eels Under a Commercial RAS
Source: Animals (Basel). 2026 Jan 21;16(2):324. doi: 10.3390/ani16020324 (PMC12837468; doi:10.3390/ani16020324)
Supplement: Supplementary file 1 [file animals-16-00324-s001.zip › animals-4076346-supplementary/Supplementary Materials. Composition of the vitamin and micro-mineral premixes..pdf]

Table S1. Composition of the vitamin premix [a].

Vitamin / Compound	Unit (per kg of diet)	Amount
Vitamin A	IU	3200
Vitamin D_3	IU	1200
Vitamin E	mg	12
Vitamin K_3	mg	4
Vitamin B1	mg	3.2
Vitamin B_2	mg	1.2
Vitamin B_6	mg	3
Vitamin B_{12}	mg	0.04
Vitamin C	mg	160
Calcium pantothenate	mg	20
Nicotinic acid	mg	40
folate acid	mg	1.9
Biotin	mg	0.15
Inositol	mg	80

[a] The premix was added at 0.40% of the diet.

Table S2. Composition of the Micro-mineral premixes (mg per kg of diet)[b].

Mineral Source	Inorganic Micro-minerals (IMM)	Coated Inorganic Micro-minerals I (CIMM I)	Coated Inorganic Micro-minerals II (CIMM II)
$Cu_2(OH)_3Cl$	12.05	12.05	6.03
$FeSO_4{\cdot}H_2O$	666.67	666.67	333.34
$MnSO_4{\cdot}H_2O$	94.34	94.34	47.17
$ZnSO_4{\cdot}H_2O$	202.89	202.89	101.45
$Ca(IO_3)_2$	2.59	2.59	1.30
Na_2SeO_3	0.89	0.89	0.45
$CoSO_4$	3.64	3.64	1.82

[b] The premixes were added at 0.10% (IMM and CIMM I) or 0.05% (CIMM II) of the diet.
